# Association Between the Korean Healthy Eating Index and High-Sensitivity C-Reactive Protein: A Nationally Representative Study in South Korea, 2022–2024

**DOI:** 10.3390/nu18203356

**Published:** 2026-10-13

**Authors:** Seong-Uk Baek, Jin-Ha Yoon

**Affiliations:** 1 Graduate School, Yonsei University College of Medicine, Seoul 03722, Republic of Korea; 2 Institute for Innovation in Digital Healthcare, Yonsei University Health System, Seoul 03722, Republic of Korea; 3 The Institute for Occupational Health, Yonsei University College of Medicine, Seoul 03722, Republic of Korea; 4 Department of Preventive Medicine, Yonsei University College of Medicine, Seoul 03722, Republic of Korea

**Keywords:** antioxidants, C-reactive protein, dietary quality, epidemiology, inflammation

## Abstract

**Background**: Maintaining favorable dietary quality may play an important role in disease prevention, and the mitigation of inflammation is considered a key mechanism. Although the association between dietary quality and inflammation has been reported, evidence from South Korea remains limited. Therefore, this study examined the association between the Korean Healthy Eating Index (KHEI) and high-sensitivity C-reactive protein (hs-CRP) levels. **Methods**: We analyzed a nationally representative sample of 14,050 adults in South Korea. The 14-item KHEI was measured on a scale of 0 to 100, with higher scores indicating better dietary quality, and was categorized into quartiles (Q1–Q4). Serum hs-CRP levels (mg/L) were categorized into low (<1), intermediate (1–<3), and high (≥3) categories. Multinomial logistic regressions were employed to determine the association of KHEI with hs-CRP levels, with the low hs-CRP group as the reference category. Odds ratios (ORs) with 95% confidence intervals (CIs) were estimated. **Results**: In the overall sample, 74.47%, 18.75%, and 6.78% of participants were classified as having low, intermediate, and high hs-CRP levels, respectively. Compared with the lowest KHEI quartile (Q1), participants in Q2, Q3, and Q4 had adjusted ORs (95% CIs) of 0.98 (0.85–1.14), 0.91 (0.78–1.05), and 0.75 (0.64–0.87), respectively, for intermediate hs-CRP levels. For high hs-CRP levels, participants in Q2, Q3, and Q4 had ORs (95% CIs) of 0.81 (0.65–1.01), 0.74 (0.58–0.93), and 0.68 (0.53–0.87), respectively, compared with Q1. **Conclusions**: Higher KHEI scores was associated with lower odds of having high hs-CRP levels. Prospective studies are needed to analyze the temporal relationship.

## 1. Introduction

Maintaining a high-quality diet is essential for the prevention and management of a wide spectrum of chronic diseases, including cardiovascular diseases and cancer [1]. Rather than focusing on individual nutrients, holistic approaches aim to capture the multifaceted nature of dietary intake and provide a more comprehensive assessment of complex dietary behaviors. The Healthy Eating Index (HEI), which was developed based on the Dietary Guidelines for Americans, is a widely used measure for assessing the multidimensional aspects of dietary quality [2]. Modeled after the U.S. HEI, the Korean Healthy Eating Index (KHEI) was developed to evaluate individuals’ adherence to the Dietary Guidelines for Koreans [3,4]. Recent epidemiological studies have shown that higher KHEI scores are inversely associated with the risks of metabolic dysfunction or mortality [5,6], supporting a potential protective role of dietary quality in promoting health among Korean adults.

Individuals with higher dietary quality are more likely to consume foods rich in vitamins and antioxidants, such as vegetables and fruits, while consuming fewer pro-inflammatory components, such as saturated fats and sugars [7,8,9], potentially contributing to lower levels of systemic inflammation. Previous studies have shown that dietary quality is inversely associated with inflammatory marker levels. For instance, the HEI was reported to be inversely associated with levels of high-sensitivity C-reactive protein (hs-CRP), interleukin (IL)-1β, and IL-6 [10,11]. Additionally, the HEI has shown inverse associations with both the systemic immune-inflammation index and the systemic inflammation response index [12,13]. Other dietary quality indices, such as the Mediterranean diet score or Dietary Approaches to Stop Hypertension score, have also been inversely associated with inflammatory markers [14].

Among the available inflammatory indicators, hs-CRP is a commonly used biomarker for assessing systemic inflammation in clinical and epidemiological settings, including risk stratification, prognostic assessment, and evaluation of treatment response [15]. Despite previous research on the link between dietary quality and hs-CRP levels, several gaps remain. Although previous studies have demonstrated associations between dietary quality indices, such as the HEI and hs-CRP [10,11], evidence on the link between dietary quality and inflammatory markers in the Korean population remains limited. To the best of our knowledge, the association of the KHEI with biomarkers of systemic inflammation, including hs-CRP, has not yet been reported. Thus, we aimed to examine the relation between the KHEI and hs-CRP levels among adults in South Korea. We hypothesized that KHEI is inversely associated with hs-CRP levels.

## 2. Methods

### 2.1. Study Population

This cross-sectional study included participants from the ninth cycle of the Korea National Health and Nutrition Examination Survey (KNHANES; 2022–2024). The Korea Disease Control and Prevention Agency (KDCA) employed a multistage clustered probability sampling design. The primary sampling units (PSUs) consisted of 576 enumeration districts in South Korea, from which approximately 25 households were selected per PSU, on average.

**Figure 1:**
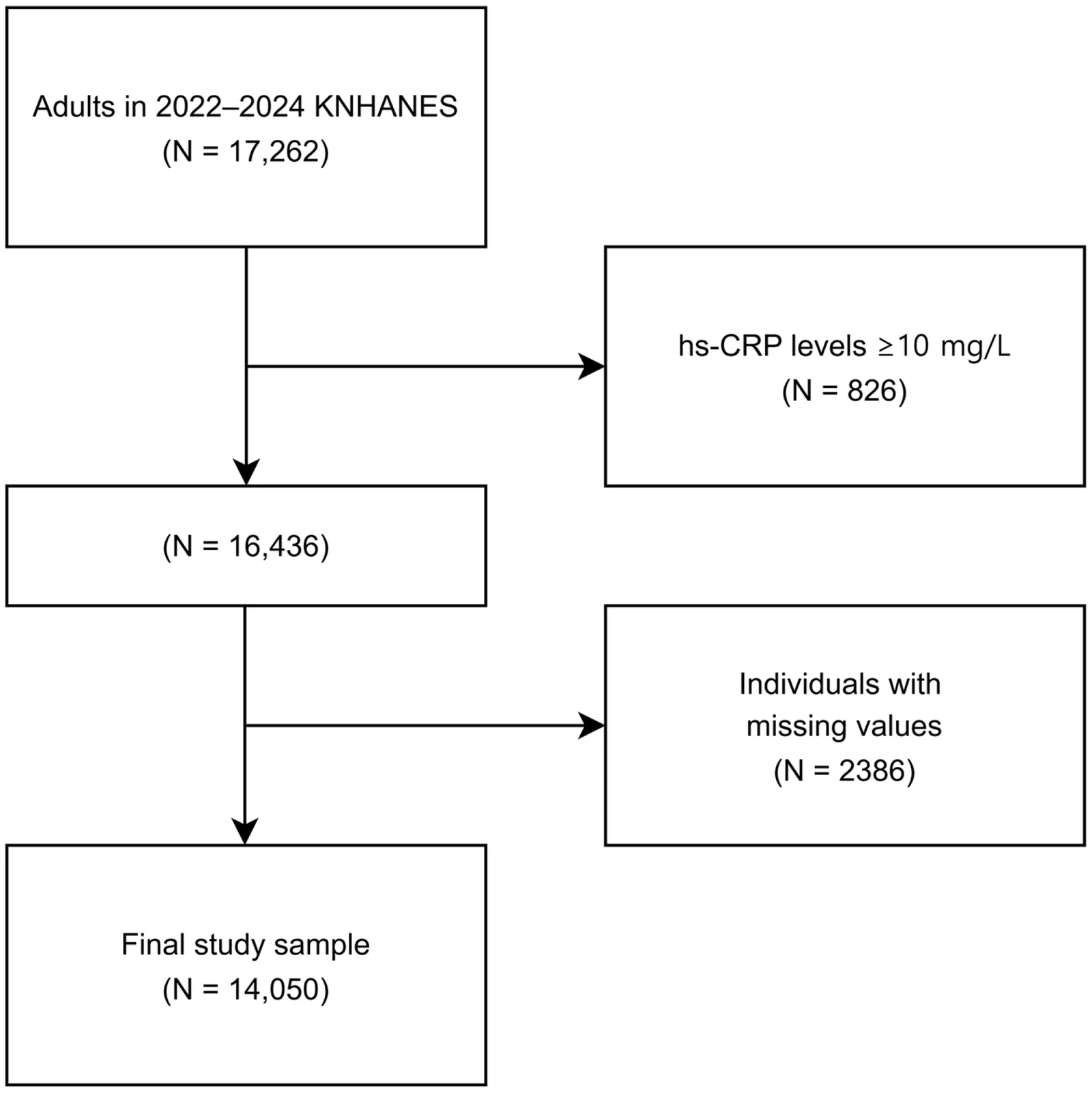
Flowchart of the sample selection procedure.

Figure 1 illustrates a flowchart of the sample selection. Initially, 17,262 individuals aged >18 years participated in the health interview, health examination, and nutrition survey components of the KNHANES 2022–2024. Subsequently, 826 participants with hs-CRP levels ≥10 mg/L were excluded because such conditions may indicate acute inflammatory status, such as infection [16,17]. After excluding individuals with missing values for variables of interest, 14,050 participants were included in the final analysis. All participants provided written informed consent. Ethical approval was granted by the Institutional Review Board of the KDCA (2018-01-03-4C-A; 2022-11-16-R-A; 2022-11-16-R-03). We adhered to the STROBE checklist for cross-sectional studies [18].

### 2.2. KHEI

The KHEI, which was developed by the KDCA, assesses the extent to which individuals adhere to the Dietary Guidelines for Koreans [3]. Nutritional assessment was conducted using a 24 h recall method, in which participants reported their food intake from the day preceding the survey, with guidance from trained dietitians to enhance the accuracy of the dietary data [3]. The KHEI consists of 14 items across three components: (1) the adequacy component, comprising eight items assessing breakfast consumption, mixed-grain intake, total fruit intake, fresh fruit intake, total vegetable intake, vegetable intake excluding kimchi and pickled vegetables, and intake of meat, fish, eggs, beans, and milk and dairy products; (2) the moderation component, comprising three items assessing intakes of saturated fatty acids, sodium, and added sugars; and (3) the energy balance component, comprising three items assessing the proportion of energy from carbohydrates, the proportion of energy from fat, and total energy intake. Further information on the KHEI items has been previously elaborated [3]. The composite KHEI score ranges from 0 to 100, with higher scores indicating better dietary quality. For the analysis, KHEI scores were categorized into quartiles based on their weighted distribution in the study sample: Q1 (lowest, <49.30), Q2 (low, 49.30–57.74), Q3 (high, 57.75–67.97, and Q4 (highest, >67.97). Continuous KHEI values were also employed in additional models.

### 2.3. hs-CRP Levels

Serum hs-CRP levels were used as the outcome variable. Blood samples were collected via venipuncture into 3 mL EDTA-coated tubes (BD Vacutainer, Franklin Lakes, NJ, USA). The samples were refrigerated at 2–8 °C and analyzed in a central laboratory within 24 h of sample collection. Serum hs-CRP levels were quantified using a particle-enhanced immunoturbidimetric assay on a Cobas 8000 analyzer (Roche, Penzberg, Germany). Values below the limit of detection (LOD, 0.2 mg/L) were imputed as LOD/√2. Following the cutoff values widely used in previous studies [16,17], hs-CRP levels (mg/L) were categorized as low (<1), intermediate (1–<3), or high (≥3). Additionally, the continuous values of hs-CRP were included as dependent variable.

### 2.4. Control Variables

A directed acyclic graph illustrating the hypothesized causal relationships between KHEI and hs-CRP is presented in Figure S1. Based on the causal diagram, the following sociodemographic and health-related variables were included as covariates in the primary models: sex, age, income (Q1, lowest to Q4, highest), education level (middle school or lower, high school, college or higher), marital status (married, unmarried or others), smoking status (no or yes), physical activity (<150 min/wk or ≥150 min/wk), and alcohol use (assessed using the Alcohol Use Disorder Identification Test-Consumption [AUDIT-C]). For further analysis, we additionally adjusted for cardiometabolic risk factors, including body mass index (BMI, kg/m^2^), diabetes mellitus (no or yes), hypertension (no or yes), and hyperlipidemia (no or yes). Considering the causal diagram (Figure S1) and previous studies supporting the mediating role of cardiometabolic risk factors [19,20], including BMI, in the association between diet and inflammation, BMI, hypertension, diabetes mellitus, and hyperlipidemia were considered as potential mediators in the primary model to prevent overadjustment and were additionally adjusted for in the supplementary analysis.

Income was categorized into quartiles based on self-reported average monthly personal income. Physical activity was categorized as no/yes according to whether participants engaged in ≥150 min/week of regular physical activity, as assessed using the Korean version of the Global Physical Activity Questionnaire [21]. Body mass index (BMI) was calculated using objectively measured height and weight. Diabetes mellitus was defined as the use of oral glucose-lowering medication or insulin, a fasting glucose level ≥126 mg/dL, or an HbA1c ≥6.5%. Hypertension was defined as a resting systolic blood pressure ≥140 mmHg, a diastolic blood pressure ≥90 mmHg, or the use of antihypertensive medication. Hyperlipidemia was defined as a total cholesterol level ≥240 mg/dL and/or the use of lipid-lowering medication.

### 2.5. Data Analysis

To determine the association of the KHEI with hs-CRP levels, multinomial logistic regression models were used, with hs-CRP grouped into three outcome categories: low, intermediate, and high. Unadjusted, sex- and age-adjusted, and fully adjusted models were sequentially fitted. Either KHEI quartiles (Q1 to Q4) or continuous KHEI values (per 10-point increase) were included as the main independent variable. Subsequently, the association between KHEI and log-transformed hs-CRP levels was examined using linear regression models, with the results presented as β coefficients (95% CI) and corresponding percentage changes (95% CI). Additionally, a potential non-linear relationship between KHEI and log-hs-CRP levels was assessed using restricted cubic splines with four knots placed at the 5th, 35th, 65th, and 95th percentiles of the KHEI distribution [22]. For further analysis, the associations between individual KHEI items and hs-CRP levels were explored. Each KHEI component was examined separately in the regression models, because correlations among the components could introduce multicollinearity. Additionally, multiple testing was not accounted for. Thus, these analyses were considered exploratory rather than confirmatory. Odds ratios (ORs) and 95% confidence intervals (CIs) were computed. For sensitivity analysis, we employed multiple imputations to address missing values and assess the robustness of our findings. Specifically, twenty complete datasets were generated based the multivariate imputation by chained equations [23]. The estimates from each dataset were then combined. For participants with missing dietary intake variables, the total KHEI score was imputed (variable name: “HEI” in the KNHANES datasets). As an additional sensitivity analysis, we included individuals who had been excluded from the primary analysis because of high hs-CRP levels (>10.00 mg/L). Finally, potential mediators of the association between KHEI and hs-CRP, including cardiometabolic risk factors such as BMI, hypertension, diabetes mellitus, and hyperlipidemia, were additionally adjusted for in the additional model. Data analysis was conducted using R for Windows (version 4.6.0). Specifically, the sampling weights (“wt_tot”), strata (“kstrata”), and primary sampling units (“psu”) were incorporated into the survey design. To pool the 2022, 2023, and 2024 datasets, the sampling weights were divided by 3, which corresponds to the number of survey cycles pooled. The complex survey design was then specified using the “svydesign” function, and the resulting survey design object was incorporated into the “svyglm” and “svy_vglm” functions for the regression analyses. Multinomial logistic regression models were fitted using the “svyVGAM” package. Linear regression models were fitted using the “svyglm” function, and the restricted cubic spline function was applied to the linear regression model using the R package “rms” and its function “rcs.” Finally, multiple imputations were conducted using the “mice” package in R.

## 3. Results

Table 1 summarizes the sample characteristics. Compared to those in the lowest KHEI quartile, participants in the higher KHEI quartiles were more likely to be female, be older, have higher income levels, have lower education levels, be unmarried, be non-smokers, have lower AUDIT-C scores, and have diabetes, hypertension, or hyperlipidemia. In the overall sample, 74.47%, 18.75%, and 6.78% of participants were classified as having low, intermediate, and high hs-CRP levels, respectively. The proportions of participants with high hs-CRP levels were 8.45%, 6.83%, 6.12%, and 5.73% among those in the Q1, Q2, Q3, and Q4 KHEI quartiles, respectively.

Table 2 shows the findings from multinomial regression models. In the fully adjusted model (Model C), compared to those in the lowest KHEI quartile (Q1), participants in Q2, Q3, and Q4 had ORs with 95% CIs for the intermediate hs-CRP level of 0.98 (0.84–1.14), 0.91 (0.78–1.05), and 0.75 (0.64–0.87), respectively. For high hs-CRP levels, the corresponding ORs (95% CIs) were 0.81 (0.65–1.01), 0.74 (0.58–0.93), and 0.68 (0.53–0.87) for participants in Q2, Q3, and Q4, respectively, compared to participants in the lowest KHEI quartile (Q1). Using the KHEI as a continuous value, the KHEI score was inversely associated with both intermediate hs-CRP levels (OR, 0.92; 95% CI, 0.89–0.96) and high hs-CRP levels (OR, 0.88; 95% CI, 0.82–0.94) in the fully adjusted model. Comparable results were observed in the sensitivity analysis using multiple imputations (Table S1). The sensitivity analysis including participants with hs-CRP ≥ 10 mg/L yielded a similar inverse association between KHEI and hs-CRP levels (Table S2). Furthermore, the estimates from the model additionally adjusted for cardiometabolic risk factors (BMI, hypertension, hyperlipidemia, and diabetes mellitus) were comparable to those from the primary analysis (Table S3).

**Figure 2:**
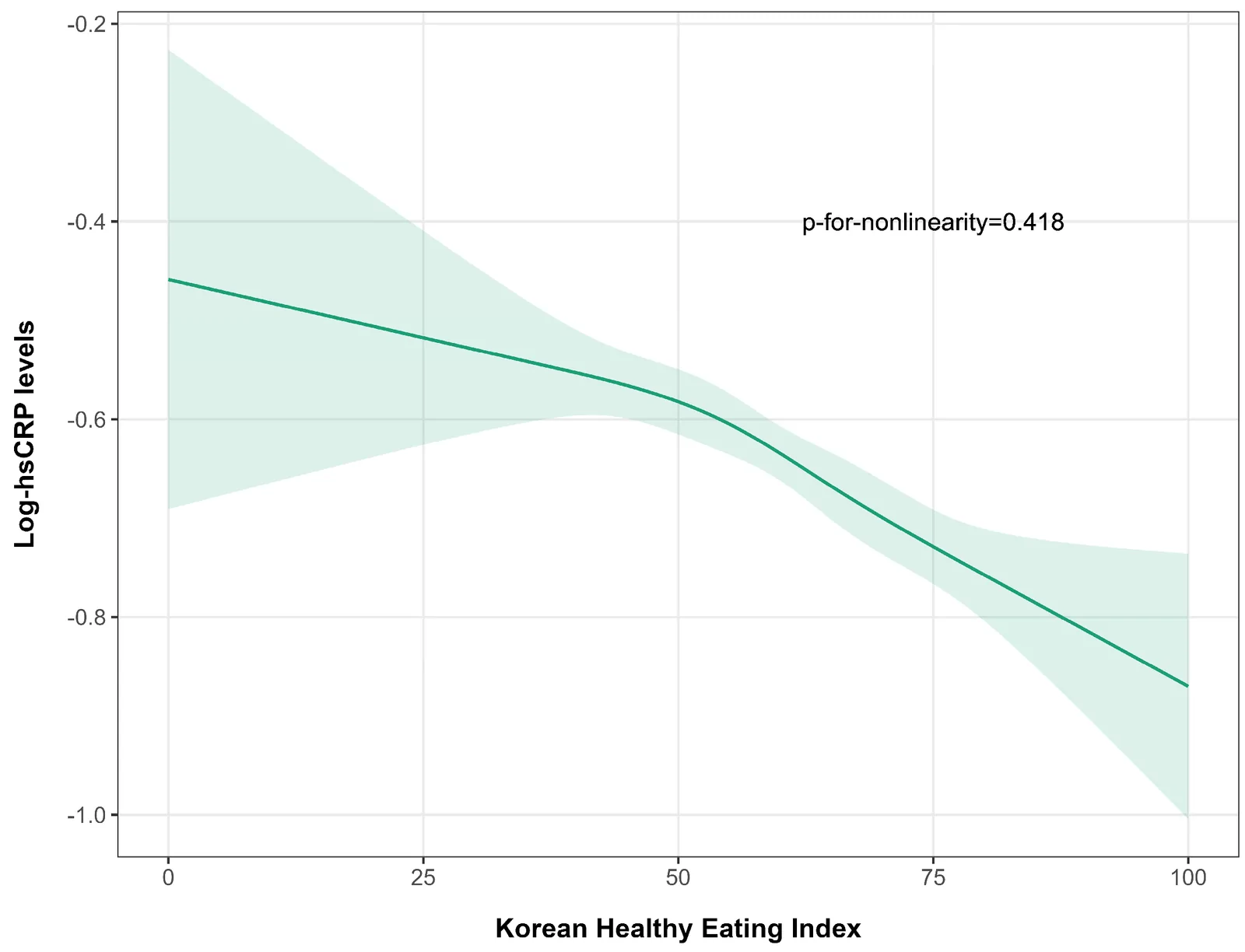
Non-linear association between KHEI and log-hs-CRP levels using fully adjusted linear regression model. The model adjusted for sex, age, income, education level, marital status, smoking, physical activity, and alcohol use. (KHEI, Korean Healthy Eating Index; hs-CRP, high-sensitivity C-reactive protein).

Table 3 shows the findings from linear regression models. In the fully adjusted model (Model C), compared to those in the lowest KHEI quartile (Q1), participants in Q2, Q3, and Q4 had % change with 95% CIs for the hs-CRP level of −5.40% (−10.69%, 0.20%), −9.67% (−14.88%, −4.13%), and −14.74% (−19.74%, −9.44%), respectively. Additionally, the non-linear association between the KHEI and hs-CRP levels in the fully adjusted model is illustrated in Figure 2. The p-for-nonlinearity was 0.418.

Table 4 presents the associations between the individual KHEI items and hs-CRP levels. Among the individual items, the total vegetable intake score was inversely associated with the odds of high hs-CRP levels (OR, 0.91; 95% CI, 0.86–0.96). Similar patterns were observed for the score for vegetable intake excluding kimchi and pickled vegetables, with ORs (95% CIs) of 0.92 (0.87–0.97) for high hs-CRP levels. In addition to vegetable intake, fresh fruit and protein-rich foods (meat, fish, eggs, and beans) were inversely associated with high hs-CRP levels.

## 4. Discussion

In this cross-sectional study involving Korean adults, we found that the KHEI score was inversely associated with hs-CRP levels. Higher dietary quality may be associated with lower levels of systemic inflammation, as reflected by hs-CRP. However, future prospective studies are needed to elucidate the temporal ordering between the KHEI and hs-CRP levels.

These findings corroborate those from previous studies showing that better dietary quality is associated with lower levels of inflammatory markers, such as hs-CRP. For example, previous cross-sectional studies have demonstrated that HEI was inversely associated with hs-CRP levels [10,11,24]. Additionally, the HEI has consistently exhibited inverse associations with other indicators of systemic inflammation, such as white blood cell count, neutrophil count, neutrophil-to-lymphocyte ratio, and systemic immune-inflammation index, among U.S. adults [12,13,25]. Among the individual KHEI items, the vegetable intake score also showed an inverse association with hs-CRP levels, consistent with previous studies [26,27,28]. Given that the KHEI shares specific items and a conceptual framework with the U.S. HEI, the inverse association with hs-CRP observed in the present study is consistent with previous evidence [2,29]. Nevertheless, our findings extend the evidence on the connection between dietary quality and systemic inflammation to a population whose eating habits are shaped by local dietary guidelines and practices, thereby moving beyond the Western-centric populations in which the HEI has traditionally been studied.

The relationship between the KHEI and low hs-CRP levels may be attributable to complex biological mechanisms. Individuals with high KHEI scores may consume greater amounts of foods, such as fruits and vegetables, which are rich in antioxidants and other anti-inflammatory components, including carotenoids, vitamins C and E, flavonoids, and dietary fiber. These dietary components may contribute to the regulation of oxidative stress and inflammatory responses through pathways involving reactive oxygen species, nuclear factor erythroid 2-related factor 2, and nuclear factor kappa B [30,31,32,33]. Additionally, greater intake of antioxidants, as reflected by higher KHEI scores, may be associated with lower circulating levels of IL-6, which is involved in hepatic synthesis of hs-CRP [34,35,36]. However, given the cross-sectional design of the present study, these biological mechanisms were not directly evaluated and should therefore be considered hypothetical pathways from previous studies rather than mechanisms demonstrated in this study.

This study has some limitations. First, the cross-sectional study design limits causal interpretation. Therefore, the observed association between the KHEI and hs-CRP should not be interpreted as indicating causal effects of the KHEI. The possibility of reverse causality should also be considered. For example, participants in the higher KHEI quartiles were older and had a higher prevalence of chronic diseases. Individuals with chronic conditions or chronic inflammation may be more likely to modify their dietary habits to promote their health. Therefore, higher KHEI scores may partly reflect dietary modifications made in response to existing health conditions, rather than long-term dietary patterns that preceded inflammatory status. Future longitudinal or interventional research is warranted to further examine the temporal relationship between the KHEI and hs-CRP levels. Second, the KHEI was assessed using the 24 h recall method, which does not accurately capture individuals’ long-term dietary habits and dietary quality. In addition, this method is susceptible to recall bias. While trained dietitians guided respondents to enhance the accuracy of dietary data [3], future studies should consider employing food frequency questionnaires or repeated assessments of dietary quality to obtain more accurate measurements. Third, while hs-CRP is a widely used biomarker for systemic inflammation, this study could not include other pro-inflammatory markers, such as IL-1β or TNF-α, owing to the lack of information. Follow-up studies are recommended to include other inflammatory biomarkers beyond hs-CRP to provide a more comprehensive understanding of the association between KHEI and inflammation. Fourth, to control potential confounders, various health-related variables, such as chronic conditions and BMI, were included as covariates in the supplementary analysis, and participants with very high levels of hs-CRP were excluded. However, this study could not account for factors such as the use of anti-inflammatory medications, occupational and environmental exposures, which may influence dietary intake and health status [37,38,39,40]. Fifth, the analysis of individual KHEI components and hs-CRP should be considered exploratory rather than confirmatory. Given the close correlations among individual KHEI components, we could not precisely disentangle the individual associations of KHEI components with hs-CRP. In addition, formal adjustments for multiple comparisons were not performed, and the possibility of chance findings should be considered.

## 5. Conclusions

Our cross-sectional study of Korean adults examined the association of KHEI with hs-CRP levels. Favorable dietary quality, as reflected by higher KHEI scores, was associated with lower hs-CRP levels. Although longitudinal research is needed to analyze the temporal ordering between the KHEI and hs-CRP, this study highlights the potential role of dietary quality in systemic inflammation in the Korean population.

## Figures and Tables

**Table 1:** Sample characteristics.

	Overall	KHEI Quartiles			
		Q1 (Lowest)	Q2	Q3	Q4 (Highest)
	N = 14,050	N = 2991	N = 3362	N = 3644	N = 4053
Sex					
Male	6046 (49.75%)	1352 (52.89%)	1521 (52.47%)	1602 (49.49%)	1571 (44.17%)
Female	8004 (50.25%)	1639 (47.11%)	1841 (47.53%)	2042 (50.51%)	2482 (55.83%)
Age					
Mean (SD)	48.73 (16.74)	38.89 (14.28)	45.69 (16.04)	51.86 (15.59)	58.45 (14.36)
Income					
Q1 (lowest)	3339 (23.29%)	851 (27.62%)	828 (23.49%)	864 (22.60%)	796 (19.44%)
Q2	3546 (25.03%)	771 (25.73%)	857 (24.93%)	942 (25.80%)	976 (23.64%)
Q3	3561 (25.56%)	702 (24.41%)	856 (26.28%)	887 (24.15%)	1116 (27.39%)
Q4 (highest)	3604 (26.13%)	667 (22.23%)	821 (25.30%)	951 (27.44%)	1165 (29.53%)
Education level					
Middle school or below	3549 (18.20%)	388 (9.00%)	763 (15.60%)	997 (20.03%)	1401 (28.19%)
High school	4661 (34.51%)	1151 (39.50%)	1089 (34.36%)	1173 (33.08%)	1248 (31.11%)
College or higher	5840 (47.28%)	1452 (51.50%)	1510 (50.03%)	1474 (46.90%)	1404 (40.70%)
Marital status					
Married	9359 (63.04%)	1496 (46.52%)	2136 (59.79%)	2644 (70.39%)	3083 (75.47%)
Unmarried or others	4691 (36.96%)	1495 (53.48%)	1226 (40.21%)	1000 (29.61%)	970 (24.53%)
Smoking					
No	12,021 (83.38%)	2324 (75.58%)	2778 (80.78%)	3154 (85.17%)	3765 (91.99%)
Yes	2029 (16.62%)	667 (24.42%)	584 (19.22%)	490 (14.83%)	288 (8.01%)
Physical activity					
<150 min/wk	7611 (50.67%)	1524 (48.23%)	1816 (50.01%)	2006 (51.79%)	2265 (52.67%)
≥150 min/wk	6439 (49.33%)	1467 (51.77%)	1546 (49.99%)	1638 (48.21%)	1788 (47.33%)
AUDIT-C					
Mean (SD)	3.81 (3.57)	4.85 (3.65)	4.21 (3.61)	3.50 (3.45)	2.67 (3.16)
Body mass index (kg/m^2^)					
Mean (SD)	24.18 (3.87)	24.29 (4.31)	24.34 (3.94)	24.20 (3.72)	23.91 (3.43)
Diabetes mellitus					
No	11,967 (87.46%)	2737 (92.41%)	2919 (89.25%)	3049 (85.82%)	3262 (82.35%)
Yes	2083 (12.54%)	254 (7.59%)	443 (10.75%)	595 (14.18%)	791 (17.65%)
Hypertension					
No	9376 (71.59%)	2355 (81.78%)	2366 (75.03%)	2310 (67.96%)	2345 (61.58%)
Yes	4674 (28.41%)	636 (18.22%)	996 (24.97%)	1334 (32.04%)	1708 (38.42%)
Hyperlipidemia					
No	9592 (71.89%)	2385 (81.78%)	2435 (75.35%)	2385 (68.52%)	2387 (61.91%)
Yes	4458 (28.11%)	606 (18.22%)	927 (24.65%)	1259 (31.48%)	1666 (38.09%)
hs-CRP (mg/L)					
Low (<1)	10,455 (74.47%)	2171 (72.37%)	2453 (73.33%)	2711 (74.73%)	3120 (77.44%)
Intermediate (1−<3)	2674 (18.75%)	581 (19.17%)	676 (19.85%)	714 (19.15%)	703 (16.84%)
High (≥3)	921 (6.78%)	239 (8.45%)	233 (6.83%)	219 (6.12%)	230 (5.73%)

AUDIT-C, Alcohol Use Disorder Identification Test-Consumption; KHEI, Korean Healthy Eating Index; SD, standard deviation; hs-CRP, high-sensitivity C-reactive protein. Values were presented as unweighted n with survey-weighted % for categorical variables and survey-weighted mean (standard deviation) for continuous variables.

**Table 2:** Associations of the Korean Healthy Eating Index (KHEI) with high-sensitivity C-reactive protein (hs-CRP) in multinomial logistic regression models (reference outcome = low high-sensitivity C-reactive protein).

	Model A	Model B	Model C
	OR (95% CI)	OR (95% CI)	OR (95% CI)
**Outcome = Intermediate hs-CRP**			
KHEI quartiles			
Q1	Reference	Reference	Reference
Q2	1.02 (0.89–1.18)	0.95 (0.83–1.10)	0.98 (0.85–1.14)
Q3	0.97 (0.84–1.12)	0.86 (0.73–1.00)	0.91 (0.78–1.05)
Q4	0.82 (0.71–0.94)	0.69 (0.59–0.80)	0.75 (0.64–0.87)
p-for-linear trend	0.004	< 0.001	0.002
KHEI score			
Continuous scale ^a^	0.95 (0.92–0.98)	0.90 (0.87–0.94)	0.92 (0.89–0.96)
**Outcome = High hs-CRP**			
KHEI quartiles			
Q1	Reference	Reference	Reference
Q2	0.80 (0.64–0.99)	0.80 (0.64–1.00)	0.81 (0.65–1.01)
Q3	0.70 (0.56–0.87)	0.71 (0.56–0.90)	0.74 (0.58–0.93)
Q4	0.63 (0.51–0.78)	0.65 (0.51–0.83)	0.68 (0.53–0.87)
p-for-linear trend	< 0.001	< 0.001	0.012
KHEI score			
Continuous scale ^a^	0.86 (0.82–0.92)	0.87 (0.81–0.93)	0.88 (0.82–0.94)

^a^ unit = per 10-point increase OR, odds ratio; CI, confidence interval; Model A: crude model; Model B: sex- and age-adjusted model; Model C: fully adjusted model controlling for sex, age, income, education level, marital status, smoking, physical activity, and alcohol use.

**Table 3:** Associations of the Korean Healthy Eating Index (KHEI) with log-transformed high-sensitivity C-reactive protein levels in linear regression models.

	Model A		Model B		Model C	
	β (95% CI)	% Change (95% CI)	β (95% CI)	% Change (95% CI)	β (95% CI)	% Change (95% CI)
KHEI quartiles						
Q1	Reference	Reference	Reference	Reference	Reference	Reference
Q2	−0.02 (−0.08, 0.03)	−2.47 (−7.88, 3.26)	−0.07 (−0.12, −0.01)	−6.40 (−11.64, −0.84)	−0.06 (−0.11, 0.00)	−5.40 (−10.69, 0.20)
Q3	−0.05 (−0.11, 0.01)	−4.78 (−10.02, 0.75)	−0.12 (−0.18, −0.06)	−11.46 (−16.52, −6.09)	−0.10 (−0.16, −0.04)	−9.67 (−14.88, −4.13)
Q4	−0.09 (−0.14, −0.03)	−8.47 (−13.24, −3.44)	−0.19 (−0.25, −0.13)	−17.33 (−22.05, −12.32)	−0.16 (−0.22, −0.10)	−14.74 (−19.74, −9.44)
KHEI score						
Continuous scale ^a^	−0.03 (−0.04, −0.01)	−2.73 (−4.12, −1.32)	−0.06 (−0.08, −0.04)	−5.75 (−7.24–−4.24)	−0.04 (−0.05, −0.03)	−3.93 (−5.32, −2.53)

^a^ unit = per 10-point increase; CI, confidence interval; Model A: crude model; Model B: sex- and age-adjusted model; Model C: fully adjusted model controlling for sex, age, income, education level, marital status, smoking, physical activity, and alcohol use.

**Table 4:** Association between individual items of the Korean Healthy Eating Index (KHEI) and high-sensitivity C-reactive protein (hs-CRP) on multinomial logistic regression models (reference outcome = low high-sensitivity C-reactive protein).

	Model A	Model B	Model C
	OR (95% CI)	OR (95% CI)	OR (95% CI)
**Outcome** **= Intermediate hs-CRP**			
**Adequacy component**			
Having breakfast	0.99 (0.98–1.00)	0.97 (0.96–0.99)	0.98 (0.96–0.99)
Mixed-grain intake	0.97 (0.95–0.99)	0.95 (0.93–0.97)	0.96 (0.93–0.98)
Total fruit intake	0.96 (0.94–0.98)	0.95 (0.93–0.97)	0.96 (0.94–0.98)
Fresh fruit intake	0.96 (0.94–0.98)	0.95 (0.93–0.97)	0.97 (0.95–0.99)
Total vegetable intake	1.02 (0.99–1.05)	0.98 (0.95–1.01)	0.99 (0.95–1.02)
Vegetable intake excluding kimchi and pickled vegetables	1.00 (0.97–1.03)	0.98 (0.95–1.00)	0.99 (0.96–1.02)
Meat, fish, eggs, and beans intake	0.99 (0.97–1.01)	0.99 (0.97–1.00)	0.99 (0.98–1.01)
Milk and milk products intake	0.99 (0.97–1.00)	0.99 (0.98–1.00)	0.99 (0.98–1.01)
**Moderation component**			
Saturated fatty acids	1.02 (1.00–1.03)	1.01 (0.99–1.02)	1.01 (0.99–1.02)
Sodium	0.98 (0.96–0.99)	0.99 (0.97–1.00)	0.99 (0.97–1.00)
Added sugars	1.02 (1.01–1.03)	1.01 (1.00–1.03)	1.01 (1.00–1.02)
**Balance component**			
% of energy from carbohydrate	0.98 (0.96–1.00)	0.98 (0.96–1.00)	0.98 (0.96–1.00)
% of energy from fat	0.99 (0.97–1.01)	0.98 (0.96–1.01)	0.99 (0.96–1.01)
Total energy intake	0.99 (0.97–1.01)	0.99 (0.97–1.01)	0.99 (0.97–1.01)
**Outcome = High hs-CRP**			
**Adequacy component**			
Having breakfast	0.99 (0.97–1.01)	1.00 (0.98–1.02)	1.00 (0.98–1.03)
Mixed-grain intake	0.96 (0.93–0.99)	0.97 (0.94–1.00)	0.97 (0.94–1.01)
Total fruit intake	0.94 (0.91–0.98)	0.96 (0.92–0.99)	0.97 (0.93–1.01)
Fresh fruit intake	0.93 (0.90–0.97)	0.95 (0.92–0.98)	0.96 (0.92–0.99)
Total vegetable intake	0.91 (0.87–0.96)	0.90 (0.85–0.95)	0.91 (0.86–0.96)
Vegetable intake excluding kimchi and pickled vegetables	0.91 (0.87–0.96)	0.91 (0.86–0.96)	0.92 (0.87–0.97)
Meat, fish, eggs, and beans intake	0.97 (0.95–1.00)	0.97 (0.94–0.99)	0.98 (0.95–1.00)
Milk and milk products intake	0.99 (0.98–1.01)	0.99 (0.98–1.01)	1.00 (0.98–1.02)
**Moderation component**			
Saturated fatty acids	1.00 (0.98–1.01)	1.00 (0.98–1.02)	1.00 (0.98–1.02)
Sodium	0.98 (0.96–1.00)	0.99 (0.97–1.02)	0.99 (0.96–1.01)
Added sugars	1.00 (0.98–1.02)	0.99 (0.97–1.01)	0.99 (0.97–1.01)
**Balance component**			
% of energy from carbohydrate	0.98 (0.95–1.02)	0.98 (0.95–1.02)	0.99 (0.95–1.02)
% of energy from fat	0.97 (0.94–1.01)	0.97 (0.94–1.01)	0.98 (0.94–1.01)
Total energy intake	0.97 (0.93–1.00)	0.97 (0.93–1.00)	0.97 (0.94–1.01)

^a^ Model A: crude model; Model B: sex- and age-adjusted model; Model C: fully adjusted model controlling for sex, age, income, education level, marital status, smoking, physical activity, and alcohol use. OR, odds ratio; CI, confidence interval.

## Data Availability

Dataset can be obtained at https://knhanes.kdca.go.kr (accessed on 3 July 2026).

## Supplementary Materials

The following supporting information can be downloaded at https://www.mdpi.com/article/10.3390/nu18203356/s1, Figure S1. A causal diagram on the relationship between the Korean Healthy Eating Index and high-sensitivity C-reactive protein; Table S1. Association between the Korean Healthy Eating Index and high-sensitivity C-reactive protein in multinomial logistic regression models (reference outcome = low high-sensitivity C-reactive protein). Sensitivity analysis using multiple imputations; Table S2. Associations of the Korean Healthy Eating Index (KHEI) with high-sensitivity C-reactive protein (hs-CRP) in multinomial logistic regression models including those with hs-CRP levels ≥ 10 mg/L; Table S3. Associations of the Korean Healthy Eating Index (KHEI) with high-sensitivity C-reactive protein (hs-CRP) in multinomial logistic regression models additionally adjusting for cardiometabolic risk factors.
