# Supplementary material for: Association Between the Korean Healthy Eating Index and High-Sensitivity C-Reactive Protein: A Nationally Representative Study in South Korea, 2022–2024
Source: Nutrients. 2026 Oct 13;18(20):3356. doi: 10.3390/nu18203356 (PMC13708019; doi:10.3390/nu18203356)
Supplement: Supplementary file 1 [file nutrients-18-03356-s001.zip › nutrients-4592629-supplementary.pdf]

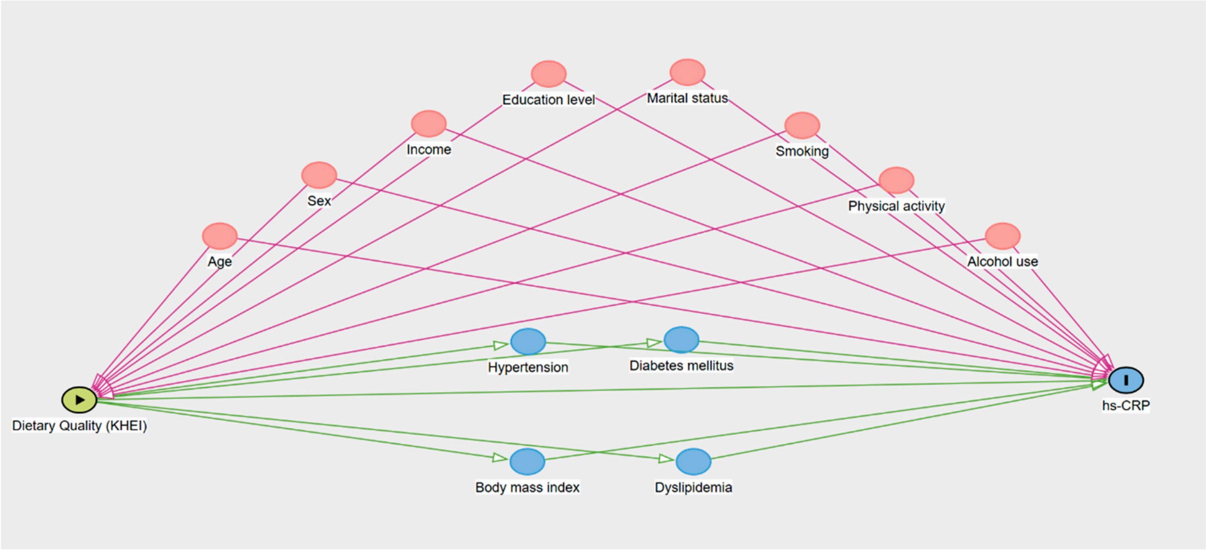

Figure S1. A causal diagram on the relationship between the Korean Healthy Eating Index and high-sensitivity C-reactive protein

Table S1. Association between the Korean Healthy Eating Index and high-sensitivity C-reactive protein in multinomial logistic regression models (reference outcome = low high-sensitivity C-reactive protein). Sensitivity analysis using multiple imputations

	**Model 1**	**Model 2**	**Model 3**
	**OR (95% CI)**	**OR (95% CI)**	**OR (95% CI)**
Outcome = Intermediate hs-CRP			
KHEI quartiles			
Q1	Reference	Reference	Reference
Q2	0.99 (0.87, 1.13)	0.92 (0.81, 1.06)	0.95 (0.83, 1.09)
Q3	0.95 (0.83, 1.09)	0.84 (0.73, 0.97)	0.89 (0.77, 1.03)
Q4	0.84 (0.73, 0.95)	0.71 (0.61, 0.82)	0.77 (0.67, 0.89)
KHEI score			
Continuous scale[a]	0.95 (0.92, 0.99)	0.90 (0.87, 0.94)	0.93 (0.89, 0.96)
Outcome = High hs-CRP			
KHEI quartiles			
Q1	Reference	Reference	Reference
Q2	0.80 (0.66, 0.98)	0.79 (0.64, 0.97)	0.81 (0.66, 0.99)
Q3	0.70 (0.57, 0.85)	0.69 (0.56, 0.85)	0.72 (0.58, 0.89)
Q4	0.62 (0.51, 0.75)	0.61 (0.49, 0.76)	0.65 (0.52, 0.81)
KHEI score			
Continuous scale[a]	0.86 (0.82, 0.91)	0.86 (0.81, 0.91)	0.87 (0.82, 0.93)

aunit = per 10-point increase OR, odds ratio; CI, confidence interval Model A: crude model; Model B: sex- and age-adjusted model; Model C: fully adjusted model controlling for sex, age, income, education level, marital status, smoking, physical activity, and alcohol use.

Table S2. Associations of the Korean Healthy Eating Index (KHEI) with high-sensitivity C-reactive protein (hs-CRP) in multinomial logistic regression models including those with hs-CRP levels ≥ 10 mg/L

	**Outcome = Intermediate hs-CRP**	**Outcome = High hs-CRP**
	**OR (95% CI)**	**OR (95% CI)**
**KHEI quartiles**		
Q1	Reference	Reference
Q2	0.99 (0.85–1.14)	0.78 (0.64–0.96)
Q3	0.91 (0.78–1.06)	0.70 (0.56–0.87)
Q4	0.75 (0.64–0.88)	0.69 (0.55–0.85)
**KHEI score**		
Continuous scale[a]	0.92 (0.89–0.96)	0.88 (0.82–0.93)

[a] unit = per 10-point increase

The model adjusted for sex, age, income, education level, marital status, smoking, physical activity, and alcohol use

Table S3. Associations of the Korean Healthy Eating Index (KHEI) with high-sensitivity C-reactive protein (hs-CRP) in multinomial logistic regression models additionally adjusting for cardiometabolic risk factors

	**Outcome = Intermediate hs-CRP**	**Outcome = High hs-CRP**
	**OR (95% CI)**	**OR (95% CI)**
**KHEI quartiles**		
Q1	Reference	Reference
Q2	0.97 (0.84–1.12)	0.81 (0.65–1.01)
Q3	0.91 (0.78–1.07)	0.75 (0.59–0.94)
Q4	0.78 (0.66–0.91)	0.73 (0.57–0.93)
**KHEI score**		
Continuous scale[a]	0.93 (0.89–0.97)	0.89 (0.83–0.96)

[a] unit = per 10-point increase

The model adjusted for sex, age, income, education level, marital status, smoking, physical activity, alcohol use, body mass index, diabetes mellitus, hypertension, and hyperlipidemia
